# 127 The Teen-Aged Life Impact Burn Recovery Evaluation Profile: Initial Development

**DOI:** 10.1093/jbcr/irae036.126

**Published:** 2024-04-17

**Authors:** Khushbu F Patel, Madeleine McGwin, Mary D Slavin, Kate Surette, Alexandra R Gladstone, Tina L Palmieri, Ludwik K Branski, Jeffrey C Schneider, Frederick J Stoddard, Michael Murphy, Lewis E Kazis, Colleen M Ryan

**Affiliations:** Shriners Hospitals for Children - Boston, Allston, Massachusetts; Shriners Children's Boston, Boston, Massachusetts; Department of Health Law, Policy and Management, Boston University School of Public Health, Boston, MA; Tulane University, New Orleans, Louisiana; Shriners' Childrens Boston, Swampscott, Massachusetts; UC Davis, Shriners Children's Northern California, Sacramento, CA; The University of Texas Medical Branch - Galveston, TX, Galveston, TX; Spaulding Rehabilitation Hospital/Harvard Medical School, Boston, MA; Harvard Medical School, Boston, Massachusetts; Massachusetts General Hospital/Harvard Medical School, Acton, Massachusetts; Massachusetts General Hospital/Shriners Children's, Boston, MA; Shriners Hospitals for Children - Boston, Allston, Massachusetts; Shriners Children's Boston, Boston, Massachusetts; Department of Health Law, Policy and Management, Boston University School of Public Health, Boston, MA; Tulane University, New Orleans, Louisiana; Shriners' Childrens Boston, Swampscott, Massachusetts; UC Davis, Shriners Children's Northern California, Sacramento, CA; The University of Texas Medical Branch - Galveston, TX, Galveston, TX; Spaulding Rehabilitation Hospital/Harvard Medical School, Boston, MA; Harvard Medical School, Boston, Massachusetts; Massachusetts General Hospital/Harvard Medical School, Acton, Massachusetts; Massachusetts General Hospital/Shriners Children's, Boston, MA; Shriners Hospitals for Children - Boston, Allston, Massachusetts; Shriners Children's Boston, Boston, Massachusetts; Department of Health Law, Policy and Management, Boston University School of Public Health, Boston, MA; Tulane University, New Orleans, Louisiana; Shriners' Childrens Boston, Swampscott, Massachusetts; UC Davis, Shriners Children's Northern California, Sacramento, CA; The University of Texas Medical Branch - Galveston, TX, Galveston, TX; Spaulding Rehabilitation Hospital/Harvard Medical School, Boston, MA; Harvard Medical School, Boston, Massachusetts; Massachusetts General Hospital/Harvard Medical School, Acton, Massachusetts; Massachusetts General Hospital/Shriners Children's, Boston, MA; Shriners Hospitals for Children - Boston, Allston, Massachusetts; Shriners Children's Boston, Boston, Massachusetts; Department of Health Law, Policy and Management, Boston University School of Public Health, Boston, MA; Tulane University, New Orleans, Louisiana; Shriners' Childrens Boston, Swampscott, Massachusetts; UC Davis, Shriners Children's Northern California, Sacramento, CA; The University of Texas Medical Branch - Galveston, TX, Galveston, TX; Spaulding Rehabilitation Hospital/Harvard Medical School, Boston, MA; Harvard Medical School, Boston, Massachusetts; Massachusetts General Hospital/Harvard Medical School, Acton, Massachusetts; Massachusetts General Hospital/Shriners Children's, Boston, MA; Shriners Hospitals for Children - Boston, Allston, Massachusetts; Shriners Children's Boston, Boston, Massachusetts; Department of Health Law, Policy and Management, Boston University School of Public Health, Boston, MA; Tulane University, New Orleans, Louisiana; Shriners' Childrens Boston, Swampscott, Massachusetts; UC Davis, Shriners Children's Northern California, Sacramento, CA; The University of Texas Medical Branch - Galveston, TX, Galveston, TX; Spaulding Rehabilitation Hospital/Harvard Medical School, Boston, MA; Harvard Medical School, Boston, Massachusetts; Massachusetts General Hospital/Harvard Medical School, Acton, Massachusetts; Massachusetts General Hospital/Shriners Children's, Boston, MA; Shriners Hospitals for Children - Boston, Allston, Massachusetts; Shriners Children's Boston, Boston, Massachusetts; Department of Health Law, Policy and Management, Boston University School of Public Health, Boston, MA; Tulane University, New Orleans, Louisiana; Shriners' Childrens Boston, Swampscott, Massachusetts; UC Davis, Shriners Children's Northern California, Sacramento, CA; The University of Texas Medical Branch - Galveston, TX, Galveston, TX; Spaulding Rehabilitation Hospital/Harvard Medical School, Boston, MA; Harvard Medical School, Boston, Massachusetts; Massachusetts General Hospital/Harvard Medical School, Acton, Massachusetts; Massachusetts General Hospital/Shriners Children's, Boston, MA; Shriners Hospitals for Children - Boston, Allston, Massachusetts; Shriners Children's Boston, Boston, Massachusetts; Department of Health Law, Policy and Management, Boston University School of Public Health, Boston, MA; Tulane University, New Orleans, Louisiana; Shriners' Childrens Boston, Swampscott, Massachusetts; UC Davis, Shriners Children's Northern California, Sacramento, CA; The University of Texas Medical Branch - Galveston, TX, Galveston, TX; Spaulding Rehabilitation Hospital/Harvard Medical School, Boston, MA; Harvard Medical School, Boston, Massachusetts; Massachusetts General Hospital/Harvard Medical School, Acton, Massachusetts; Massachusetts General Hospital/Shriners Children's, Boston, MA; Shriners Hospitals for Children - Boston, Allston, Massachusetts; Shriners Children's Boston, Boston, Massachusetts; Department of Health Law, Policy and Management, Boston University School of Public Health, Boston, MA; Tulane University, New Orleans, Louisiana; Shriners' Childrens Boston, Swampscott, Massachusetts; UC Davis, Shriners Children's Northern California, Sacramento, CA; The University of Texas Medical Branch - Galveston, TX, Galveston, TX; Spaulding Rehabilitation Hospital/Harvard Medical School, Boston, MA; Harvard Medical School, Boston, Massachusetts; Massachusetts General Hospital/Harvard Medical School, Acton, Massachusetts; Massachusetts General Hospital/Shriners Children's, Boston, MA; Shriners Hospitals for Children - Boston, Allston, Massachusetts; Shriners Children's Boston, Boston, Massachusetts; Department of Health Law, Policy and Management, Boston University School of Public Health, Boston, MA; Tulane University, New Orleans, Louisiana; Shriners' Childrens Boston, Swampscott, Massachusetts; UC Davis, Shriners Children's Northern California, Sacramento, CA; The University of Texas Medical Branch - Galveston, TX, Galveston, TX; Spaulding Rehabilitation Hospital/Harvard Medical School, Boston, MA; Harvard Medical School, Boston, Massachusetts; Massachusetts General Hospital/Harvard Medical School, Acton, Massachusetts; Massachusetts General Hospital/Shriners Children's, Boston, MA; Shriners Hospitals for Children - Boston, Allston, Massachusetts; Shriners Children's Boston, Boston, Massachusetts; Department of Health Law, Policy and Management, Boston University School of Public Health, Boston, MA; Tulane University, New Orleans, Louisiana; Shriners' Childrens Boston, Swampscott, Massachusetts; UC Davis, Shriners Children's Northern California, Sacramento, CA; The University of Texas Medical Branch - Galveston, TX, Galveston, TX; Spaulding Rehabilitation Hospital/Harvard Medical School, Boston, MA; Harvard Medical School, Boston, Massachusetts; Massachusetts General Hospital/Harvard Medical School, Acton, Massachusetts; Massachusetts General Hospital/Shriners Children's, Boston, MA; Shriners Hospitals for Children - Boston, Allston, Massachusetts; Shriners Children's Boston, Boston, Massachusetts; Department of Health Law, Policy and Management, Boston University School of Public Health, Boston, MA; Tulane University, New Orleans, Louisiana; Shriners' Childrens Boston, Swampscott, Massachusetts; UC Davis, Shriners Children's Northern California, Sacramento, CA; The University of Texas Medical Branch - Galveston, TX, Galveston, TX; Spaulding Rehabilitation Hospital/Harvard Medical School, Boston, MA; Harvard Medical School, Boston, Massachusetts; Massachusetts General Hospital/Harvard Medical School, Acton, Massachusetts; Massachusetts General Hospital/Shriners Children's, Boston, MA; Shriners Hospitals for Children - Boston, Allston, Massachusetts; Shriners Children's Boston, Boston, Massachusetts; Department of Health Law, Policy and Management, Boston University School of Public Health, Boston, MA; Tulane University, New Orleans, Louisiana; Shriners' Childrens Boston, Swampscott, Massachusetts; UC Davis, Shriners Children's Northern California, Sacramento, CA; The University of Texas Medical Branch - Galveston, TX, Galveston, TX; Spaulding Rehabilitation Hospital/Harvard Medical School, Boston, MA; Harvard Medical School, Boston, Massachusetts; Massachusetts General Hospital/Harvard Medical School, Acton, Massachusetts; Massachusetts General Hospital/Shriners Children's, Boston, MA

## Abstract

**Introduction:**

Outcome measures are critical to assess interventions, establish evidence based practice guidelines, and ultimately support the needs of teenage burn survivors’ integration into the community. Only one condition- and age-specific instrument exists to monitor burn recovery. A more granular instrument with advanced technological capability (i.e., computer adaptive test (CAT)) is needed. The current study developed a preliminary conceptual framework and item pools as first steps to create a new instrument for teen burn survivors.

**Methods:**

The International Classification of Functioning, Disability, and Health Child and Youth Version informed the conceptual framework. Initial candidate items were reviewed qualitatively through binning and winnowing. A team of experts reviewed sets of items in four rounds from March through June 2023, such that each set was reviewed twice. Experts rated each item as “keep, omit, modify, or revisit”. To identify outcomes important in assessment of recovery, focus groups of clinicians, teenagers, and parents were conducted until thematic saturation was reached. Each transcript was analyzed using deductive and inductive approaches to identify and categorize content. Findings were used to validate the framework and ensure adequate domain coverage.

**Results:**

Initial candidate items (n=5,472) from 178 generic and burn-specific assessments previously published were grouped into two domains of the conceptual framework – activity and participation. At the end of round four, experts had modified item content for 29.8% of items. Activity item pools (n=128) included mobility, physical self-care, general tasks, experience of self, and learning and applying knowledge. Participation item pools (n=73) included major life areas, interpersonal relationships, and engagement. The recall period was set to “currently”. A five-point Likert scale format for responses was assigned based on the item measurement of agreement (n=124), difficulty (n=23), or frequency (n=54) of behavior. Eighteen participants completed the study. Clinicians mean age was 48.4 years with 90% female and 80% White; teens mean age was 13.8 years with 63% female and 75% white. Burn size ranged from 0.25 to 60% TBSA. Examples of themes included body image, self-esteem, and social relationships.

**Conclusions:**

The preliminary item pool (n=201) was developed containing domains of activity and participation. The next step is to conduct cognitive interviews and refine item content prior to field-testing for the calibration and validation of the CAT.

**Applicability of Research to Practice:**

Clinical and research use of the Teen-Aged12-19 LIBRE (Life Impact Burn Recovery Evaluation) will facilitate optimization of interventions, allow for more efficient allocation of resources and care for teenagers with burn injuries.

